# AKR1C3 as a therapeutic target to overcome erlotinib resistance in lung adenocarcinoma

**DOI:** 10.1186/s40779-025-00593-4

**Published:** 2025-02-17

**Authors:** William C. Cho, Kwan P. Li, Chi F. Wong, King Y. Fung, James C. H. Chow, Ka M. Cheung, Jeffrey C. H. Chan, Eunice Y. T. Lau

**Affiliations:** 1 Department of Clinical Oncology, Queen Elizabeth Hospital, Hong Kong SAR, China; 2 Department of Obstetrics and Gynaecology, Tsan Yuk Hospital, Hong Kong SAR, China

**Keywords:** AKR1C3, Erlotinib resistance, Epidermal growth factor receptor (EGFR) mutation, Lung adenocarcinoma (LUAD), Therapeutic target

Dear Editor,

Lung adenocarcinoma (LUAD) is a major subtype of non-small cell lung cancer with global health implications. Targeted therapies, such as epidermal growth factor receptor (EGFR) tyrosine kinase inhibitors (TKIs), have demonstrated promise but encounter resistance challenges. Erlotinib (ER), a widely used EGFR TKI, often faces the emergence of resistance [1]. Therefore, understanding therapeutic targets for ER resistance is crucial. AKR1C3 plays a pivotal role as a key enzyme in the biosynthesis of androgens, serving as a regulator of hormone activity and prostaglandin F synthase. This impacts hormone receptor occupancy and cellular proliferation, thereby influencing tumor progression and drug resistance. High AKR1C3 expression has been associated with aggressive tumors, invasiveness, and poor outcomes in some cancers, potentially contributing to resistance against chemotherapeutic agents and targeted therapies [2]. Several small-molecule inhibitors have shown potent suppression of AKR1C3 function, with a few inhibitors advancing to clinical trials (e.g., ASP9521, NTC01352208 for castration-resistant prostate cancer, and BAY-1128688, NCT03373422 for endometriosis). Although no AKR1C3 inhibitor has yet received clinical approval, compelling evidence supports its potential as a promising target for pharmaceutical development. This study aims to characterize the role of AKR1C3 in LUAD cells and patients, evaluating the in vitro and in vivo therapeutic efficacy of combining ER with an AKR1C3 inhibitor in ER-resistant LUAD.

ER-resistant cells were developed from HCC827 and HCC4006 LUAD cell lines. These ER-resistant cells exhibited approximately 4-fold higher AKR1C3 expression compared with parental cells (*P* < 0.001), as confirmed by reverse transcription-quantitative polymerase chain reaction (RT-qPCR) (Fig. 1a). Knockdown of *AKR1C3* in ER-resistant cells resulted in reduced expression of stemness-associated genes, indicating its role in regulating tumor-initiating genes in LUAD (Additional file 1: Fig. S1a). RNA sequencing analysis of *AKR1C3* knockdown cells identified 585 differentially expressed genes relative to control cells (Additional file 1: Fig. S1b). Several cancer-associated genes were identified, including *CXCL5*, *PTGS2*, *SOD2*, *AMOT*, and *RPPH1*, which are involved in various crucial processes such as angiogenesis, inflammatory prostaglandin production, metastasis, DNA damage repair, proliferation, and cell cycle progression [3–5]. Gene set enrichment analysis revealed AKR1C3’s involvement in 21 gene sets and pathways, including E2F targets, G2M checkpoint, mitotic spindle, and MYC targets, providing insights into the functional mechanisms of AKR1C3 and its potential as a druggable target in LUAD (Additional file 1: Fig. S1c).

**Fig. 1:**
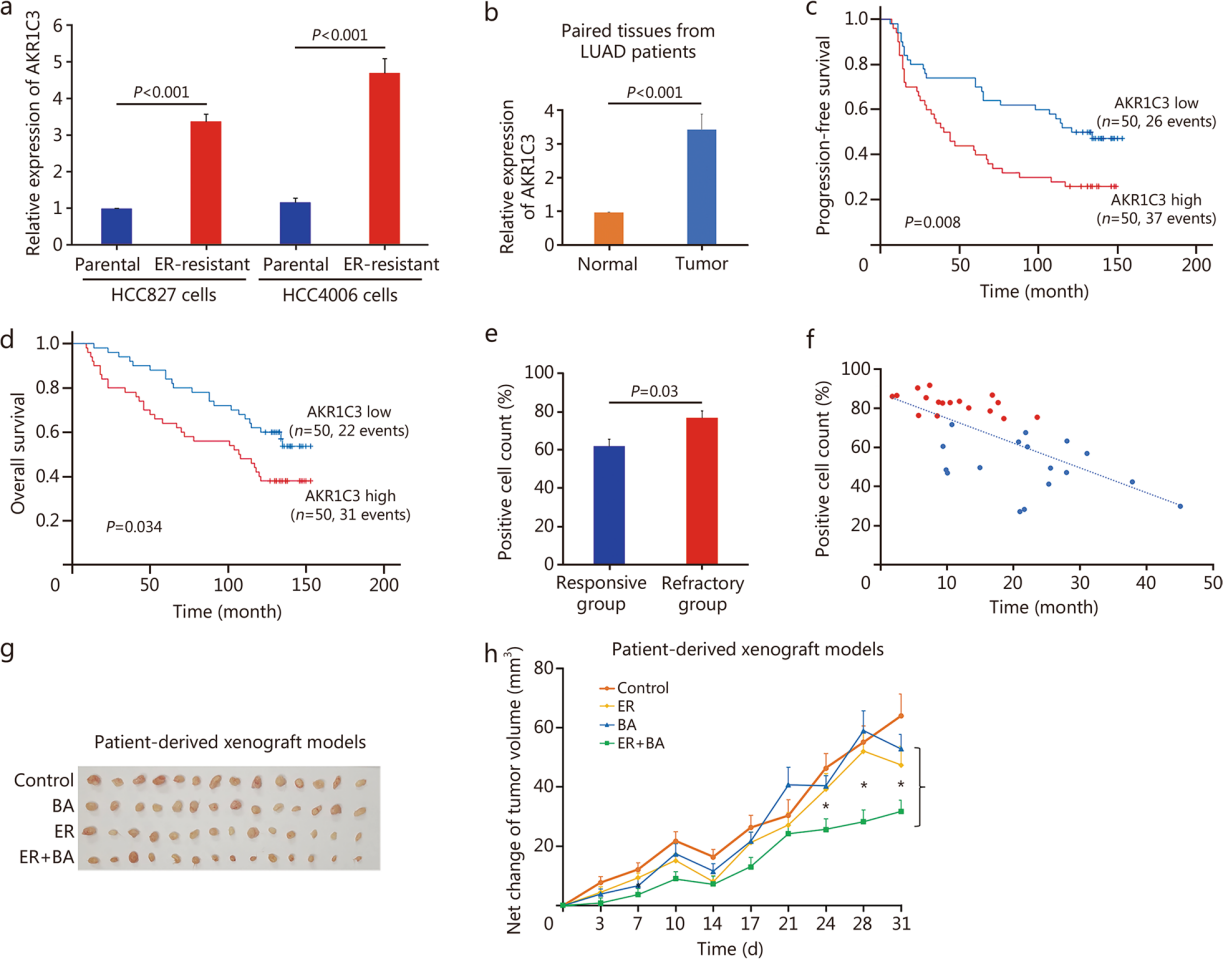
Analysis of the role of AKR1C3 in lung adenocarcinoma (LUAD) and the therapeutic potential of its inhibitor in erlotinib (ER)-resistant LUAD. **a** Relative gene expression levels of AKR1C3 were determined by reverse transcription-quantitative polymerase chain reaction (RT-qPCR) in ER-resistant cells compared to their respective parental controls (*P* < 0.001). **b** AKR1C3 gene expressions were compared between lung tumor tissues and adjacent normal tissues in a cohort of LUAD patients (*n* = 100). A significant difference was observed between normal and tumor samples (*P* < 0.001). The prognostic significance of AKR1C3 expression was determined using Kaplan–Meier analysis and log-rank test. AKR1C3 expression exhibited prognostic significance in terms of progression-free survival (**c,** *P* = 0.008) and overall survival (**d,** *P* = 0.034). **e** Another cohort of LUAD patients who received ER therapy was also included, with treatment durations up to 45 months (*n* = 58). A significant difference in AKR1C3-positive cell counts was found between the responsive (*n* = 43) and refractory (*n* = 15) groups after 1 year of ER treatment (*P* = 0.03). **f** Plot of the time taken for 34 patients to develop ER resistance against AKR1C3-positive cell counts after treatment with ER over 45 months. Red dots represent a high AKR1C3-positive cell count, while blue dots represent a low count. Pearson’s correlation coefficient (*r*) revealed a negative correlation value of −0.53 between AKR1C3-positive cell count and the days required for ER resistance development. **g** In the patient-derived xenograft model (*n* = 14 for each group), all mice were sacrificed and the tumors were excised at day 31. **h** In the patient-derived xenograft model (*n* = 14 for each group), tumor size was measured twice a week, and the net change in tumor volume was calculated. *Indicates a significant difference in tumor volume between ER group and ER + AKR1C3 inhibitor (BA) group (*P* < 0.05). The data are presented as mean ± standard error of the mean. BA 3-{[4-(trifluoromethyl)phenyl]amino}benzoic acid

We collected lung tumors and adjacent normal tissues from a cohort of 100 randomly selected LUAD patients who had undergone surgical resection at Queen Elizabeth Hospital (Hong Kong) (Additional file 1: Table S1). Measurement of AKR1C3 expression in paired lung tissues showed significant overexpression of AKR1C3 (> 3-fold) in tumor tissues compared with normal tissues (*P* < 0.001; Fig. 1b). Survival analysis revealed that high (above-median) *AKR1C3* mRNA expression was significantly associated with poorer progression-free survival (*P* = 0.008) and overall survival (*P* = 0.034; Fig. 1c, d). Tissue samples were obtained from an additional cohort of 58 ER-treated LUAD patients (Additional file 1: Table S1), aimed at establishing a correlation between AKR1C3 expression and resistance to ER therapy. The proportion of AKR1C3-positive cells was significantly higher (77% vs. 62%) in patients who developed resistance within the first year compared with those who remained sensitive (*P* = 0.03; Fig. 1e). Among the 34 patients who developed resistance after 45 months of ER treatment, a negative correlation (−0.53) was observed between the number of AKR1C3-positive cells and the time required for resistance development (Fig. 1f).

The IC50 of the AKR1C3 inhibitor 3-{[4-(trifluoromethyl)phenyl]amino}benzoic acid (BA) was determined in cancer cells to evaluate its effects on ER resistance. SynergyFinder and CompuSyn software analysis demonstrated strong synergy scores for the combination of BA and ER, with a synergy score of 12 and a combination index of 0.5. BA reduced the IC50 values of ER by 38% to 0.02 µmol/L in HCC827 cells and by 45% to 11 µmol/L in HCC4006 cells. Furthermore, AKR1C3 inhibition increased sensitivity to ER-induced apoptosis in cancer cells (Additional file 1: Fig. S2a). Combined treatment resulted in significantly higher apoptosis rates compared with ER or BA alone (Additional file 1: Fig. S2b). In cell line-derived xenograft (CDX) models, combination group exhibited significant reductions in tumor volume compared with ER group after approximately 20 d of treatment. Specifically, in the HCC827 CDX model, tumor volume was reduced by 55% (parental) and 48% (resistance) on day 32, while in the HCC4006 CDX model, the reduction was 40% (parental) and 27% (resistance) (Additional file 1: Fig. S3a, b). In the patient-derived xenograft model, the combination treatment inhibited tumor growth, leading to a significant 50% reduction in tumor volume and a 33% greater suppression of tumor weight on day 31 compared with control group (Fig. 1g, h). No significant differences in body or organ weights were observed among the treatment groups, indicating no notable toxicity.

In conclusion, AKR1C3 emerges as a promising therapeutic target for managing ER resistance in LUAD patients. The combination of an AKR1C3 inhibitor with ER represents a viable strategy to enhance treatment outcomes for patient resistant to EGFR TKI. Further research is necessary to validate the therapeutic potential of AKR1C3 and assess its efficacy in clinical trials for LUAD, offering hope for improved management of patients with ER-resistant LUAD.

## Supplementary Information

**Additional file 1.** Materials and methods. **Table S1** Demographic data of lung adenocarcinoma patients. **Fig. S1** Examination of the role of AKR1C3 in regulating tumor-initiating cell (T-IC) phenotypes and the correlation of AKR1C3 and resistance development time. **Fig. S2** In vitro effect of erlotinib (ER) and AKR1C3 inhibitor 3-{[4-(trifluoromethyl)phenyl]amino}benzoic acid (BA) co-treatment on apoptosis. **Fig. S3** Therapeutic potential of erlotinib (ER) combined with AKR1C3 inhibitor 3-{[4- (trifluoromethyl)phenyl]amino}benzoic acid (BA) in ER-resistant lung adenocarcinoma cell line-derived xenograft (CDX) models.

## Data Availability

The data supporting the findings of this study are available from the corresponding author upon reasonable request.

## Abbreviations

BA: 3-{[4-(trifluoromethyl)phenyl]amino}benzoic acid
CDX: Cell line-derived xenograft
EGFR: Epidermal growth factor receptor
ER: Erlotinib
LUAD: Lung adenocarcinoma
RT-qPCR: Reverse transcription-quantitative polymerase chain reaction
TKI: Tyrosine kinase inhibitor
