# Supplementary material for: AKR1C3 as a therapeutic target to overcome erlotinib resistance in lung adenocarcinoma
Source: Mil Med Res. 2025 Feb 17;12:8. doi: 10.1186/s40779-025-00593-4 (PMC11834314; doi:10.1186/s40779-025-00593-4)
Supplement: Supplementary file 1 — Additional file 1. Materials and methods. Table S1 Demographic data of lung adenocarcinoma patients. Fig. S1 Examination of the role of AKR1C3 in regulating tumor-initiating cell (T-IC) phenotypes and the correlation of AKR1C3 and resistance development time. Fig. S2 In vitro effect of erlotinib (ER) and AKR1C3 inhibitor 3-{[4-(trifluoromethyl)phenyl]amino}benzoic acid (BA) co-treatment on apoptosis. Fig. S3 Therapeutic potential of erlotinib (ER) combined with AKR1C3 inhibitor 3-{[4- (trifluoromethyl)phenyl]amino}benzoic acid (BA) in ER-resistant lung adenocarcinoma cell line-derived xenograft (CDX) models. [file 40779_2025_593_MOESM1_ESM.pdf]

Materials and methods

Patients

Lung tumor tissues and adjacent normal tissues were collected from a cohort of 100 randomly selected lung adenocarcinoma (LUAD) patients who had undergone surgical resection at Queen Elizabeth Hospital (Hong Kong) from 1 January 2002 to 31 December 2019. Furthermore, a separate cohort of 58 LUAD patients from the same hospital at the same time who underwent erlotinib (ER) targeted therapy was randomly selected. The duration of ER treatment for responsive patients ranged up to 45 months. Based on treatment response, these 58 patients were divided into two groups: those who developed resistance within 1 year and those who remained sensitive. Surgical specimens or biopsies were obtained during the respective operations of these patient cohorts, and this study was approved by the Queen Elizabeth Hospital's Research Ethics Committee (Kowloon Central/Kowloon East) (KC/KE-19-0286/ER-1).

RNA extraction

RNA extraction from human tissue samples and cell lines was performed using GENEzol reagent (Geneaid Biotech, Taipei, Taiwan, China) following the manufacturer's protocol. In brief, 1 ml of GENEzol reagent was added to 100 mg minced tissue or a 9 cm culture dish on ice and mixed thoroughly. Subsequently, 200 μl of chloroform was added per ml of GENEzol and vortex to ensure proper mixing. The samples were then centrifuged at 12,000 ×*g* for 15 min. The upper layer containing the RNA was carefully transferred to a new tube, followed by a 10 min-isopropanol precipitation step at room temperature. The RNA pellet was washed with 70% ethanol, air-dried, and resuspended in RNase-free water. The concentration and quality of the extracted RNA were assessed using the NanoDrop OneC Spectrophotometer (Thermo Fisher Scientific, Waltham, US).

Reverse transcription-quantitative polymerase chain reaction (RT-qPCR)

One microgram of extracted RNA was subjected to RT using superscript III (Thermo Fisher Scientific, Waltham, US) following the manufacturer's protocol. The resulting complementary DNA was then

diluted 10-fold, and 3 µl of each sample was amplified using the QuantiNova SYBR Green PCR kit (Qiagen, Venlo, Netherlands) following the manufacturer's instructions, using the LightCycler 96 instrument (Roche, Basel, Switzerland). The Ct value was calculated using the $2^{-\Delta\Delta CT}$ method, with reference to the corresponding housekeeping gene HPRT/RPL13a to obtain the relative expression levels of the AKR1C3 gene. All RT-qPCR experiments were performed with at least three technical replicates.

Immunohistochemistry

Human lung tissue blocks were sectioned into 4 µm slices and subjected to immunohistochemistry using an AKR1C3 antibody following the manufacturer's protocol (Abcam, Cambridge, UK). In brief, the samples on glass slides were dewaxed and hydrated using xylene and absolute alcohol for 10 min each. After a brief wash with running water, the samples were rinsed with Dako wash buffer (Agilent, Santa Clara, US) for 10 min. Subsequently, the samples were transferred to preheated pH 9 target retrieval solution (Tris-EDTA buffer: 10 mmol/L Tris base, 1 mmol/L EDTA solution, 0.05% Tween 20, pH 9) and incubated for 30 min at 95ºC. After cooling down and rinsing with wash buffer, endogenous enzyme activity was blocked using EnVision Flex peroxidase-blocking reagent (Agilent, Santa Clara, US) for 30 min. The samples were then rinsed with wash buffer and incubated with a 1:100 dilution of AKR1C3 primary antibodies (Abcam, Cambridge, UK) for 2 h. Following another rinse with wash buffer, the samples were incubated with HRP anti-rabbit/mouse secondary antibody (Agilent, Santa Clara, US) for 1 h. After rinsing with wash buffer, the samples were treated with DAB chromogenic substrate mix (Agilent, Santa Clara, US) for 5 min. The color development was stopped by running tap water, and the samples were stained with hematoxylin for 3 min. After a brief wash with running tap water, the samples were dehydrated using absolute alcohol and xylene for 10 min each. Finally, the samples on the slides were covered with a mounting solution. The stained slides were scanned using manual whole-slide imaging (Microvisioneer, Esslingen am Neckar, Germany) and analyzed by QuPath. QuPath was trained to identify tumor cells and quantify the AKR1C3-positive stained cells using the default settings.

Cell culture and generation of ER-resistant cell lines

Two human LUAD cell lines, HCC827 and HCC4006, both carrying *EGFR* exon 19 deletions, were purchased from the American Type Culture Collection (ATCC, Manassas, US) and cultured in a complete medium consisting of RPMI 1640 medium, 10% fetal bovine serum, and 1% penicillin-streptomycin. To establish ER-resistant cell lines, HCC827 and HCC4006 cells were chronically exposed to gradually increasing concentrations of ER hydrochloride (Medchem Express, Monmouth Junction, US) reaching up to 9 μmol/L and 3 μmol/L, respectively, over 6 months. Additionally, the human embryonic kidney cell line 293FT was purchased from Thermo Fisher Scientific and cultured according to the manufacturer's instructions.

Transcriptome sequencing analysis

A total of 4 RNA samples were extracted from HCC827 and HCC4006 cell lines, including *AKR1C3* knockdown and knockdown vector control cell lines. RNA extraction utilized GENEzol, followed by DNase treatment and cleanup using Zymo RNA Clean & Concentrator-5 (Zymo Research, Irvine, US). Extracted RNA was evaluated for quantity and quality using Qubit 3 and 2100 Bioanalyzer. Samples meeting the recommended criteria were used for total RNA sequencing, with guidelines of 10 − 100 ng RNA and a minimum RNA integrity number of 2.

Library preparation involved using 100 ng of RNA per sample, employing the Illumina Stranded Total RNA Prep, Ligation with Ribo-Zero Plus, and IDT for Illumina RNA UD Index (Illumina, San Diego, US). Libraries underwent checks using Qubit dsDNA HS Assay Kit and 2100 Bioanalyzer with High Sensitivity DNA Kit. Dilution with resuspension buffer yielded a final concentration of 4 nmol/L, and two samples were combined in a 1:1 ratio. Denaturation of the pooled library followed NextSeq 500/550 Sequencing System Guide. PhiX (Illumina, San Diego, US) was added, and sequencing utilized the NextSeq 500/550 Mid-output Kit v2.5 for 2 × 75 bp paired-end sequencing.

Data analysis involved converting the BCL file to FASTQ using bcl2fastq software and removing the T-overhang. Quality control and trimming of FASTQ files were performed with fastp. Transcript quantification was done using Salmon, and differential expression analysis utilized DESeq2. Genes with FDR less than 0.05 and absolute $\log_2$ fold change greater than 1 were considered differentially expressed.

Gene set enrichment analysis employed clusterProfiler package and Hallmark gene sets from MSigdb v7.5.1. R and Rstudio were used for DESeq2 and clusterProfiler analysis, while other tools were utilized in the Ubuntu 20.04 operating system.

Construction of the *AKR1C3* knockdown mutant

The *AKR1C3* knockdown cell line was generated using RNA interference gene silencing. The shRNA sequence targeting AKR1C3 was cloned into the pLKO.1-puro vector. Plasmid SHC002 (Sigma-Aldrich, Burlington, US) was utilized as the vector control in the experiment. The plasmids were transfected into the target cells through lentiviral transfection. The construction of lentiviral particles was performed according to the manufacturer's protocol. In brief, a mixture of 26 µl packaging mix (Sigma-Aldrich, Burlington, US), 2.6 µg vector, and 182 µl serum-free DMEM (Thermo Fisher Scientific, Waltham, US) was prepared and allowed to stand at room temperature for 15 min. The mixture was then added to the 293FT cell culture in a complete medium with 70% confluence. After 16 h of incubation at 37 °C with 5% CO_2, the medium was replaced. Following an additional 32 h of incubation, the medium was collected and centrifuged at 500 ×*g* for 5 min. The supernatant containing the viral particles was stored at − 80 °C until further use. To generate the *AKR1C3* knockdown cell lines, the viral particles were transfected into the target ER-resistant cell lines according to the Addgene protocol. In brief, 0.46 ml of viral particles in a medium was added to the target cells with 70% confluence. The cells were cultured in a complete medium supplemented with 8 µg/ml polybrene (Sigma-Aldrich, Burlington, US). After 24 h of incubation at 37 °C with 5% CO_2, the medium was replaced with fresh medium containing 1 µg/ml puromycin (Thermo Fisher Scientific, Waltham, US). The expression of AKR1C3 in the transfected cell lines was assessed using qPCR for three passages to confirm permanent transfection.

Antiproliferation assay

The antiproliferative effects of ER and an AKR1C3 inhibitor 3-{[4-(trifluoromethyl)phenyl]amino}benzoic acid (BA) were evaluated using HCC827- and HCC4006-parental and ER-resistant cancer cell lines. The cells were seeded in a 96-well plate at the desired cell

density and maintained overnight in a 37 °C CO_2 incubator. Subsequently, the cancer cells were treated with various concentrations of ER, BA, or ER + BA for a specified duration. Following the treatment, an MTS assay was performed by adding 20 μl of the MTS working solution, which consisted of a mixture of 2 mg/ml MTS reagent powder (Promega, Madison, US) and 43.8 μg/ml phenazine methosulfate (Sigma-Aldrich), to 100 μl of the cell culture in each well of the 96-well plate. The plate was then incubated at 37 °C in a CO_2 incubator for 3 h. The absorbance at 490 nm was measured using an EnSight multimode plate reader (Perkin Elmer, Waltham, US). The inhibition rate was calculated using the following equation:

$$\text{Inhibition rate} = \left(1 - \frac{\text{Absorbance of concentration X}}{\text{Absorbance of control}}\right) \times 100\%$$

The dose-response curve, as well as the inhibitory concentration IC50 values, were determined using the "Find ECanything" function in Graphpad Prism Software version 7.0 (GraphPad Software, La Jolla, US). Each experiment was conducted with a minimum of three replicates.

Annexin V-FITC apoptosis assay

The annexin V assay (BioVision, Boston, US) was conducted in accordance with the manufacturer's instructions. Briefly, 10,000 cells were seeded into a culture dish. The HCC4006 cells were treated with ER, BA, or ER+BA for 96 h. Subsequently, the medium and cells were collected and centrifuged at 500 ×*g* for 5 min. The resulting pellet was washed twice with phosphate-buffered saline. The cells were then stained with Annexin V-FITC and propidium iodide, and analyzed using the BD FACSAria™ Fusion Flow Cytometer (BD Biosciences, Franklin Lakes, US).

Cell line-derived xenograft (CDX)

Four-week-old NOD/SCID/gamma (NSG) mice of random sex were subcutaneously inoculated with 1×10^6 HCC827 or HCC4006 (parental or ER-resistant) lung cancer cells that were pre-mixed with Matrigel (Corning, New York, US) in a 1:1 ratio. Once the tumors reached approximately 0.2 cm^3 in size, the mice were orally administered drugs daily for 32 d using oral gavage. Mice were randomly divided into 4

groups (each consisting of 10 mice) and were treated with: saline as a solvent control, 15 mg/kg of ER, 80 mg/kg of BA, or a combination of 15 mg/kg ER and 80 mg/kg BA. All drugs were administered as suspensions in saline. Tumor volume was measured twice a week using the formula (long side length × short side length × short side length)/2. The net change in tumor volume was calculated by subtracting the volume on day 0. At the end of the experiment, all mice were sacrificed, and the weights of their internal organs were measured.

Patient-derived xenograft (PDX)

A male patient, aged 58, diagnosed with metastasized LUAD, exhibited the presence of L858R and T790M mutations in the *EGFR* gene, indicating resistance to ER treatment. Pleural fluid obtained from this patient was injected into NSG mice and allowed to grow for 2 m to establish a xenograft model. Subsequently, the tumors were excised and milled into small pieces using blades and further dissociated into single cells using a gentle MACS dissociator (Miltenyi Biotec, Bergisch Gladbach, Germany). The resulting cells were filtered through a 100 μm cell strainer (Corning, New York, US) and counted using trypan blue staining. A mixture of Matrigel and 5×10^5 cells (in a 1:1 ratio) was then subcutaneously injected into NSG mice (n = 14). Once the tumors reached a visible size, the mice were subsequently treated with the drugs daily for 31 d using oral gavage. Tumor size was measured twice a week by assessing the short and long dimensions. The net change in tumor volume was calculated by subtracting the volume on day 0.

Statistical analysis

All experiments were repeated a minimum of 3 times. Data analysis was carried out using GraphPad Prism Software version 7.0 and SPSS version 26. Categorical variables were described in terms of n(%), while quantitative variables were presented as the mean ± standard error of the mean (SEM) for normally distributed data and median (interquartile range) for asymmetrically distributed data. Student's two-tailed t-tests were employed to calculate P-values. Survival analysis was conducted by the Kaplan-Meier curve

and the log-rank test. The synergistic effects of combination therapy were assessed using SynergyFinder and CompuSyn software. A *P*-value < 0.05 was considered statistically significant.

Table S1 Demographic data of lung adenocarcinoma (LUAD) patients

**Characteristic**	**Cohort 1 (*n* = 100)**	**Cohort 2 (*n* = 58)**
Gender [*n*(%)]		
Male	46 (46.0)	20 (34.5)
Female	54 (54.0)	38 (65.5)
Age [years, mean (range)]	66 (35 − 83)	66 (43 − 85)
Smoking history [*n*(%)]		
Non-smoker	63 (63.0)	45 (77.6)
Smoker	37 (37.0)	13 (22.4)
Relapse status [*n*(%)]		
No or relapse ≥ 2 years	74 (74.0)	38 (65.5)
Relapse < 2 years	26 (26.0)	20 (34.5)
Overall survival [*n*(%)]		
≥ 5 years	76 (76.0)	11 (19.0)
< 5 years	24 (24.0)	47 (81.0)

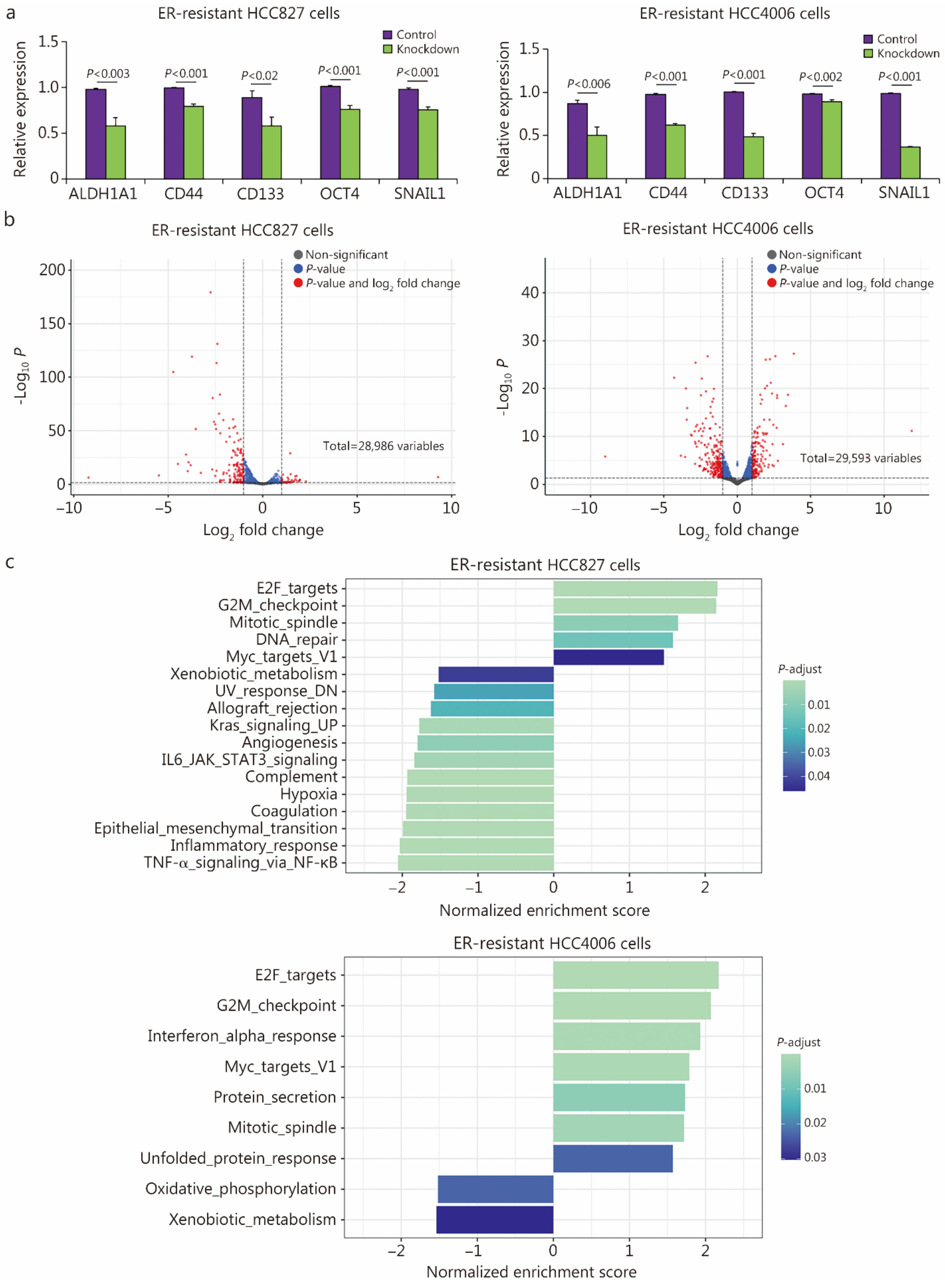

Fig. S1 Examination of the role of AKR1C3 in regulating tumor-initiating cell (T-IC) phenotypes and the correlation of AKR1C3 and resistance development time. **a** Reverse transcription-quantitative polymerase chain reaction (RT-qPCR) analysis demonstrated a significant reduction in the expression of lung T-IC markers (including ALDH1A1, CD44, CD133, OCT4, and SNAIL1) in the erlotinib (ER)-resistant HCC827 and HCC4006 cells with *AKR1C3* knockdown

compared with those without a knockdown. **b** Volcano plot depicting the differentially expressed genes (DEGs) in *AKR1C3*-knockdown HCC827 ER-resistant cells and HCC4006 ER-resistant cells compared with control cells using DESeq2 (with a $\log_2$ fold change cutoff of 1 and an adjusted *P*-value cutoff of 0.05). The grey dots represent DEGs that did not pass the adjusted *P*-value and FC cutoff. The blue dots indicate DEGs that passed the adjusted *P*-value cutoff but not the FC cutoff, while the red dots indicate those that passed both the adjusted *P*-value and FC cutoff. **c** Top gene set and pathways identified through gene set enrichment analysis (GSEA) in *AKR1C3*-knockdown HCC827 ER-resistant and HCC4006 ER-resistant cells

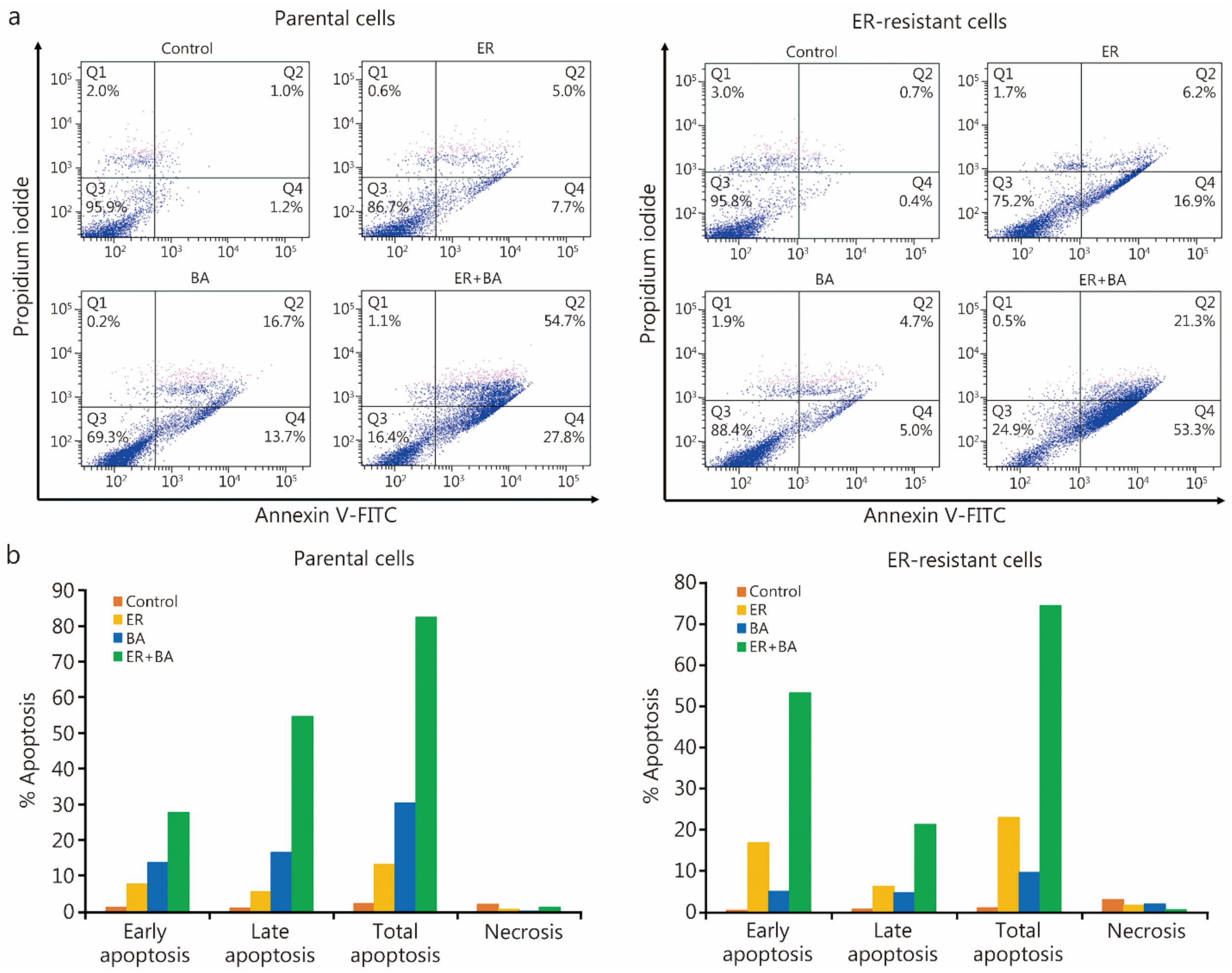

Fig. S2 In vitro effect of erlotinib (ER) and AKR1C3 inhibitor 3-{[4-(trifluoromethyl)phenyl]amino}benzoic acid (BA) co-treatment on apoptosis. **a** The apoptotic effects of control, ER, BA, and combined ER + BA treatments were evaluated using the Annexin V assay. Dot plots illustrating Annexin V-FITC/propidium iodide-stained parental and ER-resistant cells treated with the indicated drugs for 96 h are presented. **b** The percentage of apoptosis in parental and ER-resistant cells following incubation with ER and/or BA for 96 h is shown. Our findings demonstrate that the combined treatment of ER and BA enhanced the apoptotic response induced by ER, thereby increasing its efficacy as an anticancer agent

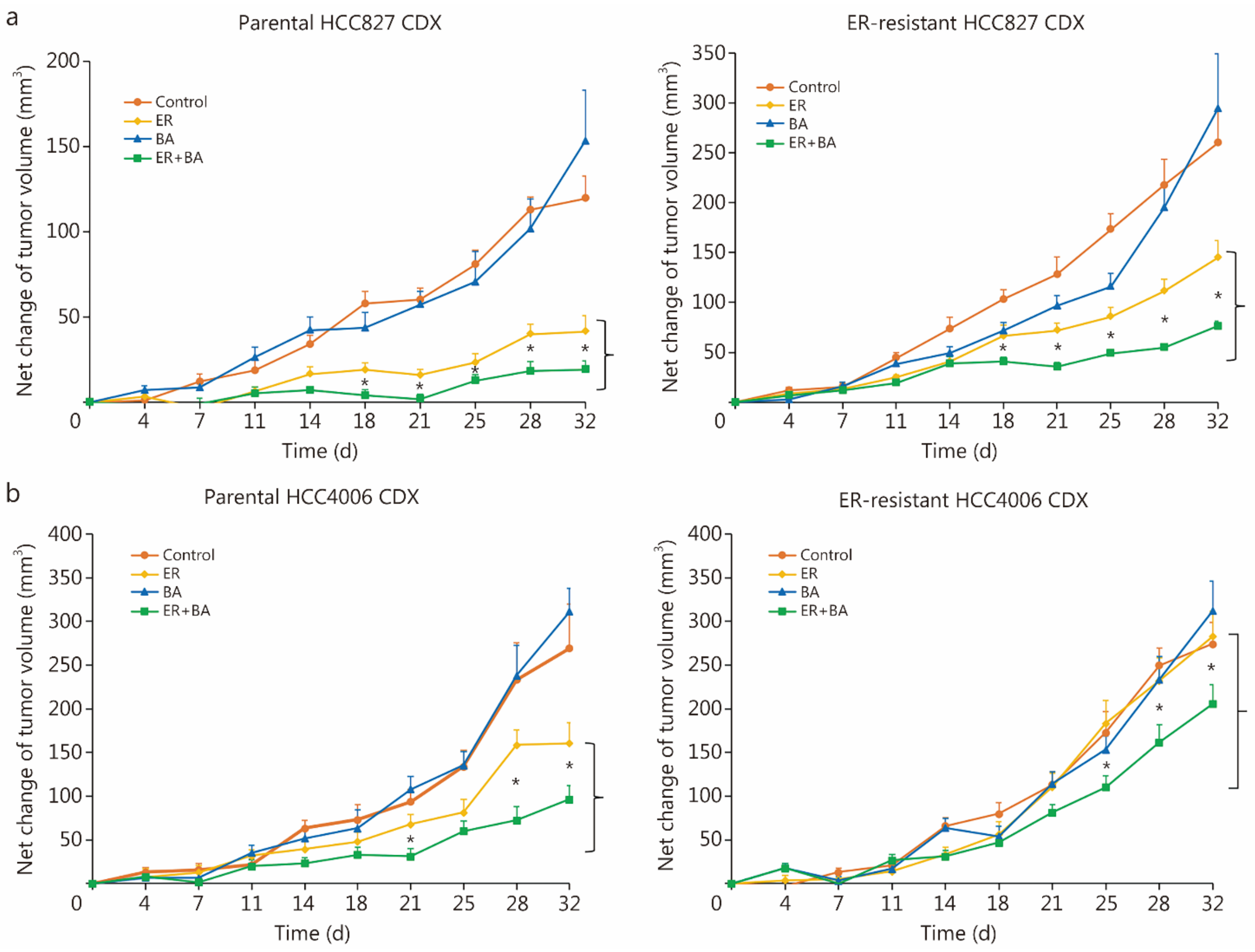

Fig. S3 Therapeutic potential of erlotinib (ER) combined with AKR1C3 inhibitor 3-{[4-(trifluoromethyl)phenyl]amino}benzoic acid (BA) in ER-resistant lung adenocarcinoma cell line-derived xenograft (CDX) models: HCC827 CDX (**a**, n = 10) and HCC4006 CDX (**b**, n = 10). CDX models were established from human cancer cells that were injected and grown in NSG mice. Once the tumors reached a visible size, the CDX were administered the drugs daily for 32 d. Tumor size was measured twice a week, and the net change in tumor volume was calculated. The tumor growth of both parental and ER-resistant HCC827 CDX and HCC4006 CDX was significantly suppressed by the combination treatment of ER with BA. *Indicates a significant difference in tumor volume between ER group and combination group (P < 0.05). The data are presented as mean ± standard error of the mean.
